# [Retracted] lncRNA DQ786243 promotes hepatocellular carcinoma cell invasion and proliferation by regulating the miR‑15b‑5p/Wnt3A axis

**DOI:** 10.3892/mmr.2024.13399

**Published:** 2024-11-18

**Authors:** Zewei Lin, Jikui Liu

Mol Med Rep 23: 318, 2021; DOI: 10.3892/mmr.2021.11957

Following the publication of this paper, and subsequently to the publication of a corrigendum (DOI: 10.3892/mmr.2022.12912) that was intended to address the issue of a pair of incorrectly written primer sequences, it was drawn to the Editors’ attention by a concerned reader that certain of the cell invasion assay data shown in Fig. 4A on p. 7 were strikingly similar to data appearing in different form in an article written by different authors at different research institutes that had already been published in the journal *Journal of Cancer*. In view of the fact that the abovementioned data had already apparently been published prior to its submission to *Molecular Medicine Reports*, the Editor has decided that this paper should be retracted from the Journal. The authors were asked for an explanation to account for these concerns, but the Editorial Office did not receive a reply. The Editor apologizes to the readership for any inconvenience caused.

